# Extracts of *Eryngium foetidum* Leaves from the Amazonia Were Efficient Scavengers of ROS and RNS

**DOI:** 10.3390/antiox12051112

**Published:** 2023-05-18

**Authors:** Deusa do Socorro Teixeira Costa Leitão, Anna Paula Pereira Barbosa-Carvalho, Francilia Campos de Siqueira, Railson Pontes e Sousa, Alessandra Santos Lopes, Renan Campos Chisté

**Affiliations:** 1 Graduate Program of Food Science and Technology (PPGCTA), Institute of Technology (ITEC), Federal University of Pará (UFPA), Belém 66075-110, Brazil; 2 Faculty of Biotechnology, Institute of Biological Sciences (ICB), Federal University of Pará (UFPA), Belém 66075-110, Brazil; 3 Faculty of Food Engineering (FEA), Institute of Technology (ITEC), Federal University of Pará (UFPA), Belém 66075-110, Brazil

**Keywords:** Amazonian plants, chlorogenic acid, antioxidant capacity, reactive oxygen species, reactive nitrogen species, green chemistry

## Abstract

*Eryngium foetidum* L. is an edible plant widespread in Amazonian cuisine and its leaves have high levels of promising phenolic compounds for the production of extracts to be used as natural antioxidant additives. In this study, the in vitro scavenging capacity of three freeze-dried extracts of *E. foetidum* leaves, obtained by ultrasound-assisted extraction using green solvents [water (H_2_O), ethanol (EtOH), and ethanol/water (EtOH/H_2_O)], was investigated against the most common reactive oxygen species (ROS) and reactive nitrogen species (RNS) generated in both physiological and food systems. Six phenolic compounds were identified, chlorogenic acid (2198, 1816 and 506 μg/g) being the major compound for EtOH/H_2_O, H_2_O, and EtOH extracts, respectively. All *E. foetidum* extracts were efficient in scavenging all the ROS and RNS (IC_50_ = 45–1000 µg/mL), especially ROS. The EtOH/H_2_O extract showed the highest contents of phenolic compounds (5781 μg/g) and showed the highest efficiency in scavenging all the reactive species, with high efficiency for O_2_^•−^ (IC_50_ = 45 μg/mL), except for ROO^•^, for which EtOH extract was the most efficient. Therefore, *E. foetidum* leaf extracts, especially EtOH/H_2_O, showed high antioxidant potential to be used as natural antioxidants in food formulations and are promising for nutraceuticals products.

## 1. Introduction

*Eryngium foetidum* L., one of the main non-conventional vegetables grown in Brazil [1] (also known as *Plantas Alimentícias Não-Convencionais*, PANCs, in Portuguese), is native to the Amazonia biome and Central America [2]. It is a vegetable widely used as a condiment in Amazonian cuisine and has attracted the attention of researchers because its use in traditional medicine to treat different pathologies and due to the presence of bioactive compounds with promising antioxidant properties [3,4,5,6,7,8,9].

Exogenous antioxidant compounds absorbed from the diet, as well as endogenous antioxidants produced by the human body, can inhibit or delay cell damage by scavenging reactive oxygen species (ROS) and reactive nitrogen species (RNS). Although ROS and RNS have beneficial physiological effects, such as cell signaling, ROS and RNS can impair the normal functioning of cells when overproduced in the physiological system as a result of oxidative stress [10]. Although the endogenous defense mechanisms of the human body, such as glutathione and the enzymatic systems superoxide dismutase, catalase, and glutathione peroxidase, are able to reduce the oxidative damage induced by the overproduction of ROS and RNS [10,11,12], endogenous antioxidants are generally not enough, and it is therefore necessary to supply antioxidants from exogenous sources.

The understanding of the action of ROS and RNS in food systems is of great importance to the food industry, since these reactive species are responsible for initiating oxidative reactions, altering the nutritional, chemical, and sensory characteristics of foods during their processing, storage, and commercialization, making them less acceptable or even unacceptable to consumers [13].

Due to the biological nature of foods, ROS and RNS are frequently formed. Studies on the chemistry of ROS and RNS and their effects on foods have been reported, since ROS are primarily responsible for initiating oxidative reaction in foods, interacting mainly with lipids, proteins, sugars, and vitamins, producing undesirable volatile compounds, degrading essential fatty acids, amino acids, and vitamins, and forming carcinogenic compounds [14,15]. Antioxidants naturally present in foods or added during processing are able to reduce and inhibit lipid peroxidation, delaying the formation of undesirable compounds responsible for oxidative rancidity and helping to maintain the nutritional quality and increase the shelf life of foods and food ingredients [16]. In addition, antioxidant compounds can be also added to minimize changes in flavor, aroma, color, or nutritional value of processed foods that might be exposed to oxidative processes.

From this perspective, to support the application of natural extracts from plants as exogenous sources of antioxidants, it is necessary to carry out systematic investigations, from initial in vitro to in vivo studies, to determine their scavenging capacities against ROS and RNS of physiological importance. As widely reported in the literature, the frequent intake of bioactive compounds with antioxidant properties demonstrates a positive association with a decrease in the risk of developing chronic-degenerative diseases, such as cardiovascular diseases [17], atherosclerosis [17,18], Alzheimer’s, and cancer [10,19].

Extracts of *E. foetidum* leaves (35–140 µg/mL) were shown to decrease intracellular ROS formation as induced by exposure to lipopolysaccharide for 24 h in a dose-dependent manner [5]. Thomas et al. [20] reported that leaf, stem, and root essential oils of *E. foetidum* leaves were able to scavenge DPPH radicals, while the essential oil of its leaves exhibited the highest reducing potential (FRAP assay). According to the literature, *E. foetidum* leaves were reported to contain phenolic acids in their composition, such as ferulic, syringic, galic, *p*-coumaric, protocatechuic, and sinapic acids [21] along with high contents of chlorogenic acid, followed by ferulic acid derivatives, and quercetin and luteolin derivatives [8].

In a previous study carried out by our research group, the leaves of *E. foetidum* showed high contents of bioactive compounds, especially phenolic compounds and carotenoids, and high in vitro antioxidant capacities against non-physiological and stable free radicals (ABTS and DPPH) and a highly reactive ROS named singlet oxygen (^1^O_2_) [8]. Another study [22] determined the antioxidant capacity of a methanolic extract rich in phenolic compounds of *E. foetidum* to scavenge other relevant ROS: superoxide radical (O_2_^•−^), hypochlorous acid (HOCl), hydrogen peroxide (H_2_O_2_), and peroxyl radicals (ROO^•^); but the phenolic composition was not assessed to help us understand the compounds responsible for the claimed properties.

Furthermore, systematic studies examining the antioxidant potential of plant extracts are of paramount importance to stimulate deeper future investigations to increase sustainable exploitation of underexploited sources of bioactive compounds. Moreover, as far as our knowledge is concerned, no information has been found concerning the antioxidant effects of characterized extracts of *E. foetidum* cultivated in the Amazonia to scavenge RNS, such peroxynitrite anion (ONOO^−^). Therefore, this work aimed to report the production of green extracts of *E. foetidum* leaves from the Amazonia with high contents of phenolic compounds, followed by the determination of their phenolic compounds composition and antioxidant capacity to scavenge relevant ROS and RNS to further suggest them as natural food additives or nutraceutical products.

## 2. Materials and Methods

### 2.1. Chemicals

Quercetin, chlorogenic acid, ascorbic acid, ethanol, methanol, methyl *tert*-butyl ether (MTBE), potassium persulfate methylene blue (MB), L-tryptophan, acetonitrile, formic acid, dihydrorhodamine 123 (DHR 123), 4,5-diaminofluorescein (DAF-2), 3-(aminopropyl)-1-hydroxy-3-isopropyl-2-oxo-1-triazene (NOC-5), hydrogen peroxide (H_2_O_2_), 30%, sodium hypochlorite solution with 4% available chlorine, β-nicotinamide adenine dinucleotide (NADH), phenazine methosulfate (PMS), nitroblue tetrazolium chloride (NBT), lucigenin, and all other analytical grade salts and solvents were purchased from Sigma-Aldrich (St. Louis, MO, USA). Ultrapure water was obtained from the Milli-Q System (Millipore Corp., Milford, MA, USA). For chromatographic analysis, samples and solvents were filtered using 0.22 and 0.45 µm nylon filtration membranes (Billerica, MA, USA).

### 2.2. Leaves of Eryngium foetidum

The leaves of *E. foetidum* were obtained from a vegetable farm in Santa Izabel do Pará, Brazil (Latitude: −1.29938, Longitude: −48.161, 1°17′58″ S, 48°9′40″ W) in July, 2017. The access to the selected plants was registered in the Brazilian National System for the Management of Genetic Heritage and Associated Traditional Knowledge (SisGen, A89EDD3). The leaves (approximately 500 g) were washed with distilled water, left to dry at room temperature (25 °C) for 2 h, frozen at −18 °C and freeze-dried (Liotop, L101, São Paulo, Brazil) at −60 °C in continuous vacuum for 24 h. The freeze-dried leaves were crushed, packaged in plastic bags under vacuum, and stored at −18 °C, protected from light exposure.

### 2.3. Extracts of Eryngium foetidum Leaves

The ultrasound-assisted method used to produce three extracts of *E. foetidum* leaves was carried out using three green solvents, as suggested elsewhere [23], with modifications. The following solvents were used: ethanol (EtOH), ethanol/water mixture (1:1, *w*/*v*) (EtOH/H_2_O), and water (H_2_O), and they were chosen considering the permissibility of residue in extracts after evaporation, following the Commission Directive 95/45/EC of the European Communities [24]. For each extract, the leaves were mixed with the solvent (1:10, *w*/*v*), and the phenolic compounds were extracted during 5 min in an ultrasound bath (Quimis, model 03350, São Paulo, Brazil) with a fixed frequency of 25 KHz, at room temperature. After the time in the ultrasonic bath, the extracts were centrifuged (Heraeus multifuge X1r centrifuge, Thermo Electron Led GMBH, São Paulo, Brazil) at 11,627.2× *g* for 5 min at room temperature, and the supernatant fraction was collected. The solid residues were subjected to seven more extractions under the same conditions and all centrifuged liquid fractions were combined to compose the extract. Then, the extracts containing EtOH and EtOH/H_2_O were dried and concentrated, respectively, under reduced pressure in a rotary evaporator (T < 38 °C), and the ethanol free extracts were frozen at −18 °C followed by freeze-drying (Liotop, L101, São Paulo, Brazil). All the freeze-dried extracts were stored at −18 °C until analysis, in amber flasks saturated with N_2_ flow. The extraction procedure was performed in triplicate (n = 3) for each solvent.

### 2.4. Phenolic Compounds Composition by HPLC-DAD

The freeze-dried extracts of *E. foetidum* leaves were solubilized in methanol/water (80:20, *v*/*v*) and injected into an Agilent HPLC (Agilent 1260 Infinity model, Santa Clara, CA, USA) equipped with a quaternary pump (G1311C), an automatic injector (G7129), an oven (G1316A), and a DAD detector (G1328C). The phenolic compounds were separated on a Synergi Hydro C_18_ column (Phenomenex, 4 μm, 250 × 4.6 mm), using a linear gradient of water/formic acid (99.5: 0.5, *v*/*v*) and acetonitrile/formic acid (99.5: 0.5 *v*/*v*) as mobile phase, according to the procedure described in detail by [25]. The UV-visible spectra were obtained between 200 and 600 nm, and chromatograms were processed at 270, 320, and 360 nm. The phenolic compounds were quantified by external standards, and the limits of detection (LOD) and quantification (LOQ) were calculated using the parameters of the analytical curves [26]. The six-point analytical curves (3.12–100 μg/mL, in duplicate) were quercetin (360 nm, R^2^ = 0.99, LOD = 0.15 μg/mL and LOQ = 0.62 μg/mL), luteolin (320 nm, R^2^ = 0.99, LOD = 0.15 μg/mL and LOQ = 0.62 μg/mL), and chlorogenic acid (320 nm, R^2^ = 0.99, LOD = 0.15 μg/mL and LOQ = 0.62 µg/mL). The contents of phenolic compounds were expressed in μg/g of extract (dry basis), considering three independent extraction procedures (n = 3). The phenolic compounds were tentatively identified by combining the following information: elution order and retention time on the C_18_ column, comparison with authentic standards analyzed under the same conditions, and UV-Visible spectra in comparison with the same compounds previously identified and quantified by our research group by mass spectrometry using electrospray as ionization source and sequential fragmentation data (HPLC-DAD-ESI-MS^n^) [8].

### 2.5. In Vitro Antioxidant Capacity of E. foetidum Extracts to Scavenge ROS and RNS

Except for the ^1^O_2_-quenching assay, all the other assays were carried out in a microplate reader (Synergy HT, BioTek, Winooski, VT, USA), equipped with a thermostat, by fluorescence, absorbance, or chemiluminescence measurements. No interference were observed among the extracts at the highest tested concentration for each assay with the specific probes. Chlorogenic acid, quercetin, and ascorbic acid were used as positive controls. Each ROS/RNS-scavenging assay corresponds to four independent experiments, each performed in triplicate (n = 3), at six different concentrations of *E. foetidum* extracts.

The results were expressed as percentage of inhibition (mean ± standard error of the mean, SEM) and the extract concentrations that were able to decrease the effect of each ROS and RNS by 50% (IC_50_) were obtained from the curves of the percentage of inhibition versus antioxidant concentration using Origin Pro v8 software (OriginLab Corporation, Northampton, MA, USA).

#### 2.5.1. Singlet Oxygen (^1^O_2_)-Quenching Assay

The ^1^O_2_-quenching capacity of *E. foetidum* extracts was determined by monitoring the inhibition of L-tryptophan degradation during ^1^O_2_ generation by photosensitization of MB [8]. Five concentrations of *E. foetidum* extracts (125 to 1000 µg/mL) were tested, along with quercetin (0.78 to 4.75 µg/mL) and chlorogenic acid (0.78 to 4.75 µg/mL) as positive controls. Kinetic data obtained from the intensity of tryptophan absorbance decay (219 nm) were fitted to a first-order reaction (Equation (1)), using the Origin Pro 8 software and the rate constants were calculated (Equation (2)). The percentage of protection that *E. foetidum* extract (EXT) (or positive controls) offered to tryptophan (TRP) was calculated using Equation (3).

(1) $$Y=Y\infty +A.\exp^{\left(-k.x\right)}$$

(2) k=ln2t12

(3) $$\mathrm{Protection}\;\left(\%\right)=\frac{k_{obs}^{\mathrm{TRP}}-k_{obs}^{\mathrm{TRP}+\mathrm{EXT}}}{k_{obs}^{\mathrm{TRP}}}\times 100$$

where Y is the TRP absorbance; Y∞ is the TRP absorbance at infinite time; A is the pre-exponential factor; *k* is the pseudo-first order rate constant; x is the reaction time; t_1⁄2_ is the half-life time (min); $k_{obs}^{\mathrm{TRP}}$ is the observed pseudo-first order rate constant fitted to the TRP decay curve (obtained in the blank experiment without the antioxidant); and $k_{obs}^{\mathrm{TRP}+\mathrm{EXT}}$ is the observed pseudo-first order rate constant fitted to the TRP decay curve in the presence of *E. foetidum* extracts.

#### 2.5.2. Superoxide Radical (O_2_^•−^)-Scavenging Assay

O_2_^•−^ was generated by the NADH/PMS/O_2_ system and the O_2_^•−^-scavenging capacity was determined by monitoring the effect of the *E. foetidum* extracts (or positive controls) on the NBT reduction induced by O_2_^•−^ at 560 nm, after 10 min of incubation at room temperature [23]. The concentrations of the *E. foetidum* extracts varied from 3.91 to 125 µg/mL; for quercetin it was from 0.001 to 30 µg/mL, and for chlorogenic acid it was from 12.5 to 250 µg/mL. The results were expressed as percentage inhibition of the reduction of NBT to formazan.

#### 2.5.3. Hypochlorous Acid (HOCl)-Scavenging Assay

The HOCl-scavenging capacity of *E. foetidum* extracts was measured by monitoring the inhibition of the increase in fluorescence resulting from the oxidation of DHR 123 to rhodamine 123 as induced by HOCl [23]. The fluorescence measurement was observed at the excitation wavelength at 485 ± 20 nm and emission at 528 ± 20 nm. HOCl was prepared by adjusting the pH of a NaOCl solution 1% (*w*/*v*) to 6.2 with the dropwise addition of 10% H_2_SO_4_, and the concentration was determined by spectrophotometry at 235 nm (ε = 100 M^−1^·cm^−1^). The *E. foetidum* extracts were tested from 31.5 to 650 µg/mL, quercetin from 0.001 to 30 µg/mL, and chlorogenic acid from 0.24 to 7.81 µg/mL.

#### 2.5.4. Hydrogen Peroxide (H_2_O_2_)-Scavenging Assay

The H_2_O_2_-scavenging capacity was determined by monitoring the effect of *E. foetidum* extracts on the inhibition of chemiluminescence observed during the H_2_O_2_-induced oxidation of lucigenin [23]. The readings were carried out after 5 min of reaction in the presence of *E. foetidum* extract (3.91 to 125 µg/mL), quercetin (0.001 to 30 µg/mL), or chlorogenic acid (12.50 to 250 µg/mL).

#### 2.5.5. Peroxyl Radicals (ROO^•^)-Scavenging Assay

The evaluation of ROO^•^-scavenging capacity was determined as described elsewhere [23] and measured by monitoring the effect of *E. foetidum* extracts on the inhibition of fluorescence decay of fluorescein resulting from ROO^•^-induced oxidation. The reaction media was pre-incubated at 37 °C for 15 min. The fluorescence signal was monitored every minute at the emission wavelength at 528 ± 20 nm, with excitation at 485 ± 20 nm, until total fluorescence decay. Trolox was used as positive control. The protection provided by the antioxidants was calculated using the difference between the area under the fluorescence decay curve in the presence of antioxidants and in its absence. The ROO^•^-scavenging capacity was expressed as the ratio between the slope of each extract (or positive control) and the slopes obtained for Trolox curves, as previously described [27].

#### 2.5.6. Peroxynitrite Anion (ONOO^−^)-Scavenging Assay

The ONOO^−^-scavenging capacity was determined by monitoring the effect of *E. foetidum* extracts on the inhibition of the increase in fluorescence resulting from the oxidation of DHR 123 to non-fluorescent rhodamine 123 as induced by ONOO^−^ [23]. ONOO^−^ was chemically synthesized as described elsewhere [28]. The fluorescence measurement was monitored at the excitation wavelength at 485 ± 20 nm and emission at 528 ± 20 nm, after 2 min of incubation at 37 °C. Parallel tests were carried out in the presence of 25 mM NaHCO_3_ to simulate physiological conditions. The *E. foetidum* extracts were tested from 400 to 900 µg/mL, and chlorogenic acid from 0.06 to 1.95 µg/mL.

### 2.6. Statistical Analysis

All the results (mean ± standard deviation or standard error of the mean) were calculated and submitted to analysis of variance (one-way ANOVA) and the means were compared by Tukey’s test, or Student’s test, at the significance level of 95% (*p* < 0.05) with Statistica 8.1(Statsoft) software.

## 3. Results and Discussion

### 3.1. Composition of Phenolic Compounds in the E. foetidum Extracts

**Figure 1:**
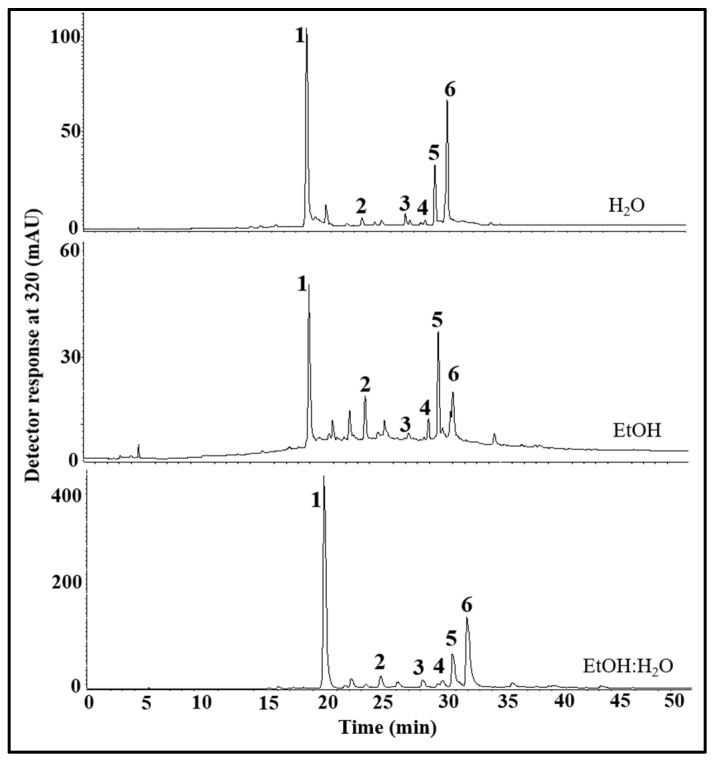
HPLC-DAD chromatogram of phenolic compounds from *Eryngium foetidum* leaf extracts. The peak characterization is given in Table 1. Peaks: 1—chlorogenic acid; 2—feruloylquinic acid; 3—quercetin glucuronide; 4—luteolin hexoside; 5—luteolin glucuronide; 6—ferulic acid derivative.

HPLC-DAD analysis of the freeze-dried extracts of *E. foetidum* leaves allowed the separation (Figure 1) and quantification (Table 1) of six phenolic compounds.

The phenolic compounds composition showed that chlorogenic acid was the major phenolic compound in all the extracts (Table 1), accounting for 38% (EtOH/H_2_O), 45% (EtOH) and 53% (H_2_O) of the identified compounds, and confirming another previous report for *E. foetidum* leaves [8].

Chlorogenic acid is a biologically active phenolic compound and is one of the phenolic acids widely found naturally in certain plant species, such as *Camelia sinensis*, also being one of the main phenolic compounds of coffee. Chlorogenic acid has versatile properties as it acts both as a nutraceutical and food additive. As a nutraceutical, it exhibits antioxidant, anti-inflammatory and anti-hypertensive properties, among others, which can contribute to the prevention and treatment of several associated pathologies [29,30,31,32]. On the other hand, as food additive, chlorogenic acid inhibits lipid oxidation, prevents degradation of other bioactive compounds, has antimicrobial activity, and can act as prebiotic [17,29].

Among the tested extracts, the EtOH/H_2_O extract presented the highest contents of phenolic compounds (5581 µg/g extract), followed by the H_2_O extract (3416 µg/g extract). The sum of the identified phenolic compounds in the EtOH extract (1104 µg/g extract) was three and five times lower than the total contents observed for the H_2_O and EtOH/H_2_O extracts, respectively (Table 1). The differences among the contents found in the extracts were expected and are commonly associated in the literature with the polarities of the studied solvents, which modulate the type of extracted compounds based on the affinity with the solvent [33,34,35,36,37,38]. Furthermore, depending on the food matrix, the combination of two or more solvents during extract preparation may allow the extraction of higher amounts of certain types of phenolic compounds than isolated solvents [39,40,41]. In addition, the extraction of different classes of phenolic compounds considering the use of solvents with different polarities has been widely discussed in the literature [42,43,44,45].

**Figure 2:**
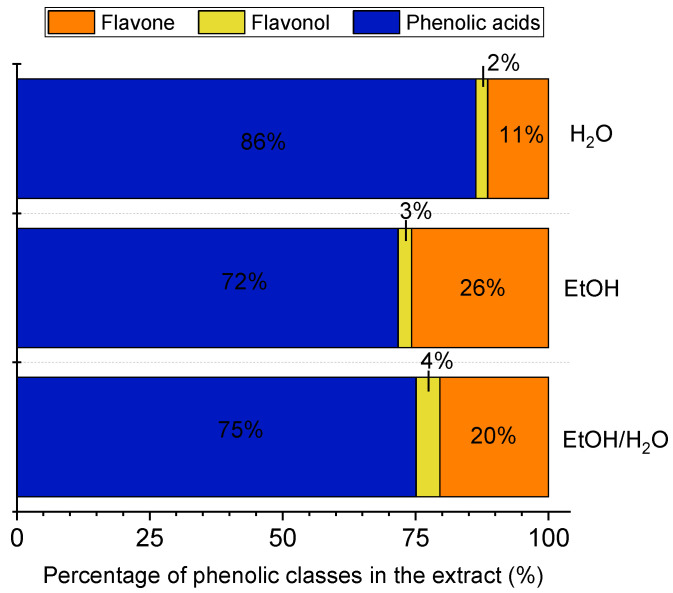
Percentage of the phenolic classes in the extracts of *Eryngium foetidum* leaves as obtained by ultrasound-assisted extraction using water (H_2_O), ethanol (EtOH), or a mixture of ethanol/water (1:1, *v*/*v*) (EtOH/H_2_O) as green solvents.

According to our study (Figure 2), the highest efficiency to extract the most polar phenolic compounds (phenolic acids) was observed for H_2_O, which has the highest polarity among the solvents. On the other hand, flavones were better extracted with EtOH (the lowest polarity), and the addition of water decreased the ability of ethanol to solubilize this type of phenolic compounds. Interestingly, the mixture of water and ethanol yielded the highest contents of flavonols, being represented in our study by quercetin glucuronide (Table 1).

### 3.2. In Vitro Antioxidant Capacity of Eryngium foetidum Extracts to Scavenge ROS and RNS

In general, all the extracts of *E. foetidum* leaves were able to inhibit the oxidizing effects of the tested reactive species (Table 2) in a concentration-dependent manner, except for the H_2_O extract, which did not show quenching ability at the highest tested concentration (1000 µg/mL).

Among the extracts, the EtOH/H_2_O presented the highest antioxidant efficiency, probably due to its high contents of phenolic compounds, mainly phenolic acids (≈75% of the total sum) (Table 1). Recently, phenolic acids have received special attention due to their relevant pharmacological contributions associated with improvement of health as consequence of their antioxidant and anti-inflammatory properties, providing protection against diseases related to oxidative stress [17,30,46]. In addition to physiological effects, phenolic acids were also reported as efficient antioxidants in food systems [47].

The EtOH/H_2_O extract showed superior efficiency in quenching ^1^O_2_ (IC_50_ at 76 μg/mL) as compared to the EtOH extract (IC_50_ at 1000 μg/mL), while the H_2_O extract did not show any antioxidant activity at the highest tested concentration (1000 µg/mL). The quenching efficiency observed for the EtOH/H_2_O extract of *E. foetidum* was 3.5 times higher than *S. diploconos* extract (IC_50_ = 269 μg/mL) [48] and close to the values reported for *Caesalpinia crista* leaf extracts (IC_50_ = 61 μg/mL) [49], but much lower than chlorogenic acid and quercetin (Table 2). ^1^O_2_ can generate alkyl radicals (R^•^), alkoxyl radicals (RO^•^), ROO^•^, organic hydroperoxides (ROOH), O_2_^•−^, H_2_O_2_, hydroxyl radical (OH^•^), and ONOO^−^ [50,51,52,53,54], and cause deleterious biological effects [52,55,56,57]. In foods, ^1^O_2_ oxidizes amino acids and vitamins, yet it reacts very quickly with polyunsaturated fatty acids and aromatic amino acids due to the high reactivity of π electrons of double bonds; the greater the unsaturation of fatty acids, the higher the reaction rate with ^1^O_2_ [15]. Thus, natural extracts with bioactive compounds with high ^1^O_2_-quenching capacity are highly desirable as promising natural ingredients for food formulations to increase the stability of foods.

Regarding O_2_^•−^, the EtOH/H_2_O extract of *E. foetidum* leaves presented the highest antioxidant capacity (IC_50_ = 45 μg/mL) (Table 2), which was 1.9 and 2.3 times superior when compared to the EtOH and H_2_O extracts, respectively, but less efficient than chlorogenic acid (20 μg/mL) and quercetin (12.9 μg/mL). Conversely, Souza et al. [22] reported no antioxidant activity in a methanolic extract of *E. foetidum* at the highest tested concentration of 4 mg/mL. However, this extract exhibited similar antioxidant efficiency to walnut leaf extracts (*Ruglans regia*), whose IC_50_ was 47 μg/mL [58], and superior efficiency to *Vismia cauliflora* leaf extract (51 μg/mL) [59].

Among the ROS, O_2_^•−^ is considered an important precursor of a variety of other highly reactive pro-oxidant species [10]. O_2_^•−^ can undergo dismutation, spontaneously generating H_2_O_2_ at low pH, or through enzymatic catalysis (superoxide dismutase, SOD), and most of the H_2_O_2_ generated is used in the formation of HOCl, which is 100 to 1000 times more toxic than O_2_^•−^ [60]. Thus, bioactive compounds able to scavenge O_2_^•−^ can be used to delay oxidative processes, including lipid peroxidation, which is one of the main pathways to food deterioration during processing and storage [11].

Concerning HOCl, the EtOH/H_2_O extract was about twice as effective as compared to the EtOH and H_2_O extracts. When compared to the literature, this *E. foetidum* extract was more efficient than *Caesalpinia crista* leaf extracts (IC_50_ at 170 μg/mL) [49]. However, Souza et al. [22] reported higher antioxidant efficiency in the methanolic extract of *E. foetidum* (13 μg/mL) than the extracts obtained in our study (IC_50_ from 118 to 222 μg/mL). The importance of being an efficient HOCl-scavenger is mainly due to the fact that this ROS, by itself, is considered a strong oxidant that reacts with O_2_^•−^ to generate ^•^OH, another highly reactive ROS known as a potent pro-inflammatory agent [61], and which also decreases food stability as consequence of lipid peroxidation [15].

In relation to H_2_O_2_, statistic differences (*p* > 0.05) were observed among the three extracts, and the EtOH/H_2_O extract showed once more its high scavenging capacity, at approximately 2.5 to 3.5 times higher than the EtOH and H_2_O extracts, respectively. Importantly, the EtOH/H_2_O extract (IC_50_ at 110 μg/mL) was as effective as chlorogenic acid (IC_50_ at 100 μg/mL) in scavenging H_2_O_2_, yet almost three times less efficient than ascorbic acid (Table 2). The EtOH/H_2_O extract of *E. foetidum* showed higher antioxidant capacity for scavenging H_2_O_2_ than leaf extracts of *Castanea sativa* and *Quercus robur* (IC_50_ at 410 and 251 μg/mL, respectively) [62], leaf extracts of *Vismia cauliflora* (IC_50_ at 289 μg/mL), a medicinal plant species abundant in the Amazonia [59], and leaf extracts of *Meyna spinosa*, a medicine plant from India (IC_50_ at 127 μg/mL) [63]. Contrasting again, Souza et al. [22] stated that no antioxidant activity was observed in the methanolic extract of *E. foetidum* at the highest tested concentration of 4 mg/mL. H_2_O_2_ is a reactive species capable of crossing cell membranes and oxidizing various cellular compartments, the source of many of its toxic effects [64]. In foods, H_2_O_2_ can indirectly cause lipid peroxidation, because H_2_O_2_ is also a precursor of ^•^OH [15].

Contrasting the results, the EtOH extract was the most efficient extract for scavenging ROO^•^ radicals, almost twice as efficient as the H_2_O extract and trolox. The H_2_O extract presented the same capacity to scavenge ROO^•^ as observed for trolox, while the EtOH/H_2_O extract showed the least scavenging efficiency. As ROO^•^ are widely known to be generated during lipid peroxidation of unsaturated fatty acids, independently of their localization, either as part of cell membranes or in food sources and formulations, these ROS have high physiological and food relevance. When compared to the literature, the EtOH extract showed higher antioxidant capacity (1.75) than hydroalcoholic extracts of artichoke leaves (*Cynara cardunculus* L.) (0.31) [65], and extracts of pulp (0.053), peels (0.72), and seeds (0.068) of *Bactris setosa* fruits [66]. Our results certainly highlight the promising potential of *E. foetidum* extracts to be added in food formulations to extend the shelf life of processed products by delaying lipid peroxidation.

Previous studies have already pointed to the high efficiency of chlorogenic acid in protecting cells against oxidative damages caused by H_2_O_2_, ¹O_2_, and O_2_^•−^, clearly demonstrating its ability to scavenge ROS [67,68], which gives support to stimulate further deep research to understand the contribution of *E. foetidum* plants as a bioactive compound source.

Concerning RNS, Table 3 shows that all the extracts of *E. foetidum* leaves were able to inhibit the oxidative/nitrosative effects of the tested RNS in a concentration-dependent manner, but with much lower efficiency than the positive controls. To the best of our knowledge, this is the first study reporting the RNS-scavenging capacity of *E. foetidum* extracts.

Either in the presence or absence of NaHCO_3_, the EtOH/H_2_O extract presented the highest ONOO^-^-scavenging capacity, while EtOH and H_2_O extracts showed similar IC_50_ values with no statistical difference (Table 3). In the experiments with the presence of NaHCO_3_ to simulate the presence of CO_2_ in the physiological system, the antioxidant capacity tended to decrease as the IC_50_ values increased. Overall, the *E. foetidum* extracts were less efficient in scavenging RNS than ROS (Table 2).

By comparison, the extracts of *Vismia cauliflora* leaves, a medicinal plant also from the Amazonia, presented a much higher ONOO^-^-scavenging capacity (5.8 μg/mL) [59] than the *E. foetidum* extracts, while gallic acid (IC_50_ at 876 μg/mL) was only 1.6 times more efficient [49]. As chlorogenic acid showed a very high antioxidant efficiency (IC_50_ at 0.23 μg/mL), either in the presence or absence of NaHCO_3_, it is probable that other non-identified compounds in the *E. foetidum* extracts might have interfered with the observed antioxidant effect.

Peroxynitrite is one of the most potentially harmful RNS, and it is generated in vivo by the reaction of nitric oxide (^•^NO) with O_2_^•−^. ONOO^−^ can undergo homolysis of the O−O bond, generating extremely reactive ^•^NO_2_ and ^•^OH [69,70]. The formation of ^•^OH by ONOO^−^ homolysis is approximately one million times faster than the metal-catalyzed generation of ^•^OH from H_2_O_2_ via the Fenton reaction [71]. Another highly relevant factor is that ONOO^−^ participates in one- and two-electron oxidation reactions with lipids, proteins and DNA, playing a key role in the process of cell/tissue injury under pathological conditions [14]. These factors together justify systematic further research to uncover natural efficient scavengers of such relevant RNS.

## 4. Conclusions

The study clearly demonstrated that extracts of *E. foetidum* leaves showed efficient antioxidant capacity to scavenge biologically relevant ROS and RNS. The EtOH/H_2_O extract presented the highest phenolic compound contents, highlighting chlorogenic acid as the major compound, and, in general, the higher antioxidant performance. Our results suggested that the *E. foetidum* extracts have promising compounds to be used in food formulations to delay lipid peroxidation, or even for drug design purposes to efficiently scavenging ROS during oxidative stress.

Therefore, this study stated the importance of further systematic research on the biological properties of *E. foetidum*, which can be seen as a promising Amazonian plant to be rationally exploited by sustainable means for obtaining natural antioxidants.

## Figures and Tables

**Table 1:** Chromatographic information and contents of phenolic compounds in the freeze-dried extracts of *Eryngium foetidum* leaves obtained by the green solvents.

Peaks	t_R (min)_ ^2^	λ_max_ (nm) ^3^	Phenolic Compound *	Concentration (μg/g Extract) ^1^		
				EtOH/H_2_O	EtOH	H_2_O
1	18.5–19.1	300(sh), 326	Chlorogenic acid ^4^	2198 ± 38 ^a^	506 ± 32 ^b^	1816 ± 160 ^a^
2	25.1–25.7	265, 326	Feruloylquinic acid ^4^	169 ± 9 ^a^	120 ± 10 ^b^	121 ± 1 ^b^
3	27.2–27.7	354	Quercetin glucuronide ^5^	258 ± 5 ^a^	28 ± 4 ^c^	77 ± 12 ^b^
4	28.6–28.8	265,300(sh), 347	Luteolin hexoside ^6^	225 ± 74 ^b^	36 ± 3 ^c^	373 ± 52 ^a^
5	29.5–29.7	265, 347	Luteolin glucoronide ^6^	956 ± 20 ^a^	248 ± 5 ^b^	17 ± 8 ^c^
6	30.9–31.1	285(sh), 330	Ferulic acid derivative ^4^	1976 ± 43 ^a^	166 ± 5 ^c^	1012 ± 115 ^b^
			Sum of compounds	5781 ± 191 ^a^	1104 ± 50 ^c^	3416 ± 348 ^b^

^1^ n = 3 (dry basis). ^2^ Retention time on C_18_ Synergi Hydro column (4 μm). ^3^ Solvent: gradient of 0.5% formic acid/water and acetonitrile/0.5% formic acid. * Peak assignment was based on the UV-visible spectra and chromatographic features observed for the same plant, previously reported by our research group, as identified by LC-MS [8]. The peaks were quantified as ^4^ chlorogenic acid, ^5^ quercetin and ^6^ luteolin equivalents. Same superscript letters in the line showed no significant difference (*p* < 0.05)

**Table 2:** Antioxidant capacity of *Eryngium foetidum* extracts to quench singlet oxygen (^1^O_2_), and to scavenge hypochlorous acid (HOCl), hydrogen peroxide (H_2_O_2_), superoxide radical (O_2_^•−^), and peroxyl radicals (ROO^•^).

Extract	IC_50_ (μg/mL) ^1^				
	^1^O_2_	O_2_^•−^	HOCl	H_2_O_2_	ROO (S_sample_/S_Trolox_) ^2^
EtOH/H_2_O	76 ± 7	45 ± 3 ^b^	118 ± 9 ^b^	110 ± 2 ^c^	0.23 ± <0.01 ^d^
EtOH	50.0 * ± 0.3	87 ± 5 ^a^	222 ± 1 ^a^	279 ± 12 ^b^	1.75 ± <0.01 ^b^
H_2_O	NA	103 ± 3 ^a^	221 ± 4 ^a^	400 ± 3 ^a^	1.00 ± <0.01 ^c^
**Positive Control**					
Chlorogenic acid	2.1 ± 0.1 ^a^	20 ± 2 ^c^	1.2 ± 0.1 ^d^	100 ± 4 ^c^	25.00 ± <0.01 ^a^
Quercetin	1.9 ± 0.1 ^a^	12.9 ± 0.5 ^d^	14 ± 1 ^c^	NA	1.34 ± <0.07 ^e^
Ascorbic acid	ND	ND	ND	40 ± 5 ^d^	ND
Trolox	ND	ND	ND	ND	1

^1^ IC_50_ = inhibitory concentration, in vitro, to decrease by 50% the oxidizing effects of reactive species in the tested medium (mean ± standard error of the mean, n = 4). ^2^ S_sample_ = slope of the *E. foetidum* extract curves. S_Trolox_ = slope of the trolox curve. * Percentage of scavenging capacity at the highest tested concentration (1000 μg/mL). NA: no activity up to the highest tested concentration. ND: not determined. Same superscript letters on the column showed no significant difference (*p* < 0.05).

**Table 3:** Antioxidant capacity of *Eryngium foetidum* L. to scavenge peroxynitrite anion (ONOO^−^).

Extract	IC_50_ (μg/mL) ^1^	
	Absence of NaHCO_3_	Presence of NaHCO_3_
EtOH/H_2_O	537 ± 1 ^b^	596 ± 4 ^b^
EtOH	663 ± 9 ^a^	704 ± 1 ^a^
H_2_O	675 ± 6 ^a^	783 ± 2 ^a^
**Positive control**		
Chlorogenic acid	0.23 ± 0.01 ^c^	0.23 ± 0.01 ^c^
Quercetin	0.010 ± <0.002 ^d^	0.010 ± <0.006 ^d^

^1^ IC_50_ = inhibitory concentration, in vitro, to decrease by 50% the oxidizing effects of reactive species in the tested medium (mean ± standard error of the mean, n = 4). Same superscript letters on the column showed no significant difference (*p* < 0.05).

## Data Availability

The data presented in this study are available in the article.
